# Pure Air in Churches

**Published:** 1890-02

**Authors:** 


### Pure Air in Churches.

Probably all church-goers have at one time or another experienced the irresistible tendency to drowsiness or somnolence that begins to be felt about the beginning of the sermon, and is only finally dissipated on quitting the church for the open air. Many people are inclined to assume rather hastily that pulpit oratory is to be held accountable for the creation of the soporific influences of the hour; but medical men and others who have considered the subject must be aware that, in nine cases out of ten, it is the closeness and heat of the atmosphere, and not the length of the sermon that is at fault. Because churches are, as a rule, large and roomy edifices, architects assume that ventilation is unneeded, and vicars and rectors are content to hold the same belief, although they are even greater sufferers by the foul state of atmosphere than the congregation. Clergyman's sore throat, hoarseness, and voicelessness are directly induced by the constant and continued efforts of speech in a heated and relaxing atmosphere, and the faculties of the congregation are dulled and blunted by the same cause. Church windows are not made to open; and, even if they were, unless the entering air is directed upwards to a considerable height, it falls upon the heads of the congregation, and complaints of draughts are made to the church-wardens, which promptly secure the closing of the windows. Most churches are heated by stoves or hot-water coils, but in very few cases is there any arrangement for admitting fresh air to come into contact with the heated surfaces of pipes or stoves before passing into the church. Exhaust ventilators in the roof are practically unknown in churches; consequently, the foul and heated air never escapes, and after service as the heated air cools it descends, and a fresh congregation rebreathes the used air of its predecessors. In this respect churches are even worse off than theatres, where the cubic space per head is far less, for all theatres have sunlight burners in the roof of the auditorium, which act very efficiently as exits for foul air. Although different systems commend themselves to different persons, we are inclined to advocate, in winter, the admission of fresh air warmed by contact with hot water coils beneath gratings in the floor, and numerous exhaust ventilators in the roof provided with rings of gas-jets to keep up the temperature of the escaping air. In summer fresh air should be admitted by revolving panes in the windows so as to secure an upward direction, the exhaust ventilators being also kept in action. If places of worship were adequately ventilated, "church headache" would become as little known as "theatre headache" now is, thanks to the regulations that the latter places of amusement are now subjected to.—*British Med. Journal.*

